# *PMP22* gene diagnostic testing in Southwest Finland in 2005–2020. An observational, register-based study

**DOI:** 10.1007/s10048-026-00930-2

**Published:** 2026-08-31

**Authors:** Pii M. Pankakoski, Mika H. Martikainen

**Affiliations:** 1 Clinical Neurosciences, Turku University Hospital, University of Turku, Turku, Finland; 2 Neurocenter, Turku University Hospital, Turku, Finland; 3 Research Unit of Clinical Neuroscience, Neurology, University of Oulu, Oulu, Finland; 4 Neurocenter and Medical Research Center, Oulu University Hospital, Oulu, Finland

**Keywords:** PMP22, CMT1, HNPP, Genetic testing

## Abstract

Pathogenic variants of *PMP22* cause Charcot-Marie-Tooth disease type 1 A (CMT1A) and hereditary neuropathy with liability to pressure palsies (HNPP). As CMT1A is the most common inherited neuropathy and the most common pathogenic *PMP22* variants are duplications and deletions, focused analysis of this gene remains relevant in the era of large gene panels and exome studies. We investigated the use and findings of *PMP22* gene analyses carried out at Turku University Hospital (TUH; Turku, Finland) in 2005–2020. Using the electronic medical record system at TUH, we identified all patients who underwent *PMP22* genetic diagnostic testing in 2005–2020. Data on testing indication, clinical features, relevant family history, genetic testing results, and final neurological diagnoses were collected. Out of total 244 tests, 64 (26%) provided a diagnostic finding. The diagnostic yield was 33% for *PMP22* deletions that were the most common variants (58%), and 23% for *PMP22* duplications. Highest yield (45%) was in patients with both clinical presentation and family history suggestive of a genetic neuropathy. The yearly diagnostic incidence of *PMP22* related neuropathies during the study period was 0.89/100 000; 0.6/100 000 for *PMP22* deletions and 0.4/100 000 for *PMP22* duplications. In clinical diagnostic use of the *PMP22* gene analyses, the majority of diagnostic findings were related to HNPP rather than CMT1A. We suggest that HNPP merits more research attention.

## Introduction

PMP22 gene coding the peripheral myelin protein 22 is the gene most commonly associated with Charcot-Marie-Tooth disease (CMT), also known as hereditary sensory and motor neuropathy (HMSN) [1, 2]. The most common subtype of CMT is CMT1A, an autosomal dominant demyelinating neuropathy caused by a 1.5 Mb duplication of chromosome 17p11.2 containing the *PMP22* gene [3]. In addition, deletions of the same locus are associated with hereditary neuropathy with liability to pressure palsies (HNPP), an autosomal dominantly inherited neuropathy. The less common *PMP22* point mutations can result in both CMT and HNPP [3–5].

The prevalence of CMT is typically reported as 1:2500 and of CMT1A associated with *PMP22* has been described as 1:3800 to 1:12500 [4]. The epidemiology of HNPP and disease associated with *PMP22* point mutations are less well understood. The prevalence of HNPP has been reported as 7.3–16:100 000 worldwide [4, 6].

Diagnostics for CMT disorders and HNPP are based on the clinical presentation, family history, electrophysiological evaluation, and genetic testing. Typical clinical presentation of CMT includes sensorimotor polyneuropathy with predominantly distal weakness, sensory loss, and foot deformities [1, 7]. The average age of onset is for CMT mainly in the first two decades and for HNPP in the second or third decade. Neither affects the life span [4, 8].

The neurophysiological categorisation of autosomal dominant CMT is based on the degree of decrease in nerve conduction velocities. In the demyelinating CMT1, median or ulnar motor conduction velocities (CVs) are < 35 m/s, and in the axonal CMT2, CVs are > 45 m/s. Also, an intermediate CMT type, with CVs at 35–45 m/s, has been proposed [1, 7, 9]. HNPP is an autosomal dominant disease of peripheral nerves with a clinical phenotype of recurrent mononeuropathies at compression sites or resulting from apparently trivial traumas, without alternate acquired cause, with or without underlying neuropathy. The clinical variability in HNPP is large. In electrophysiological studies increase in distal motor latencies and focal motor slowing at entrapment sites can be seen [4, 10].

Overall, the genetic diagnostics of peripheral neuropathies including CMT has changed considerably with the increasing availability of next generation sequencing (NGS) technology. Gene panels designed for the diagnostics of inherited neuropathies as well as whole exome and even whole genome sequencing have resulted in a continuous increase in novel genes and variants associated with CMT [11, 12]. However, because of the central role of the *PMP22* gene in CMT1 as well as fact the deletion and duplication variants are not detected by typical NGS panels and exome sequencing, the focused analysis of the *PMP22* remains relevant [12].

We decided to investigate the use and findings of genetic analyses of the *PMP22* gene that were carried out at Turku University Hospital (TUH; Turku, Finland) in 2005–2020. TUH is a tertiary level academic hospital that provides the advanced neurological and genetic diagnostic services for a population of Southwest Finland. Our purpose was to determine the clinical characteristics of the investigated individuals, to evaluate the rate of diagnostic findings in the *PMP22* genetic tests, and to estimate the rate and spectrum of new genetic diagnoses during this period in Southwest Finland.

## Materials and methods

### Study design

We searched the electronic medical records (EMRs) of TUH for all patients that had undergone any of the genetic tests used to analyse the *PMP22* gene between 1.1.2005 and 31.12.2020. The neurology and clinical genetics EMR data of the identified patients were reviewed. Basic demographic information (gender, age at the time of testing, indication for testing, relevant family history, final neurological diagnosis when available), results of the genetic tests, and final ICD-10 neurological diagnoses were collected. Descriptions of clinical status by the referring physician were scrutinised. Formal family pedigree data or data regarding possible familial relationships among the individual patients were not available in this study. Patients with no available relevant clinical data or missing data on genetic tests were excluded from further analyses.

Positive family history was defined as a patient-reported confirmed CMT or HNPP diagnosis within two generations. It was also recorded if there were no known confirmed diagnoses in the family, or the family history was considered unclear or unknown.

The results of the genetic tests were classified as either diagnostic (positive) when a pathogenic variant that explained the symptoms of the patient was detected and non-diagnostic (negative) when such a result was not found. Subjects were also divided into three groups according to the indication for testing: (1) diagnostic testing for a clinical condition, (2) predictive testing for suspected hereditary neuropathy because of family history (defined as diagnosis of the tested disease in the family within two generations), or (3) testing because of both clinical manifestation as well as suggestive family history (defined as a family member having either similar neurological symptoms to those of the patient, or a confirmed diagnosis).

### Genetic testing methods

Genetic testing was conducted at the diagnostic genetic laboratory at TUH. Multiplex ligation-dependent probe amplification (MLPA) was utilized when testing for copy number variants (CNV) of *PMP22*. MLPA remains the preferred test for *PMP22* duplication identification [12]. Depending on the clinical condition and suspected diagnosis, either MLPA identifying *PMP22* duplication related to CMT1 or MLPA identifying *PMP22* deletion related to HNPP was requested. Only the presence of duplication or deletion was reported. If neither were found, the next-generation sequencing (NGS) of *PMP22* gene could be performed. With this method, single nucleotide variants, small < 50 bp indel variants, and copy number variants larger than 2 exons could be identified. The NGS studies were performed by various commercial providers of diagnostic genetic panel services in Finland and elsewhere in the European Union (EU). Reported variants were classified as pathogenic, likely pathogenic, and of uncertain significance according to the American College of Medical Genetics and Genomics (ACMG) criteria [13].

### Regional population data for yearly incidence and prevalence estimates

Based on the identified genetic diagnoses of CMT1A and HNPP between 2005 and 2020, the minimum estimates for the population prevalence and incidence of new diagnoses were calculated. The prevalence date was 31.12.2020. The population of the province of Southwest Finland on the prevalence date was 481 403. For incidence estimates for the time period between 1.1.2005 and 31.12.2020, the mean of the annual population figures for the period (469 000) was used (Statistics Finland, https://pxdata.stat.fi/PxWeb/pxweb/en/StatFin/StatFin__vaerak/statfin_vaerak_pxt_11re.px/).

## Results

### Demographics

A total of 244 *PMP22* genetic tests were performed to 228 patients (106 female) (Table 1). There were altogether 64 diagnostic findings, 67 genetically confirmed diagnoses of *PMP22* neuropathies, and 69 of the tests identified a variant of *PMP22* gene. The highest diagnostic yield (33%) was observed in the test for *PMP22* deletion. The test for *PMP22* duplication was the most commonly performed: 116 tests performed to 115 patients (52 female). Here, 27 tests (23%) provided a diagnostic finding. 13 of the 17 NGS tests including *PMP22* were performed to patients with a negative result from tests for *PMP22* copy number.

**Table 1:** Use of genetic tests for *PMP22* associated disease

Test	*N*	Age range (mean)	Diagnostic findings, *N* (%)	Positive family history, *N* (Y/*N*/U)
*PMP22* duplication	116	1–86 (47)	27 (23)	50 / 50 / 16
*PMP22* deletion	111	3–84 (41)	37 (33)	34 / 46 / 31
NGS tests that include *PMP22*	17	3–64 (43)	0 (0)	0 / 9 / 8
Combined	244	1–86 (44)	64 (26)	84/ 105/ 55

Positive family history refers to patient reported confirmed diagnosis of CMT or HNPP in the family within two generations. Y = yes. N = no. U = unclear

The mean age of patients who tested positive for the *PMP22* duplication was 36 years (range 1 to 82 years; *p* < 0.0043), and 42 years for the *PMP22* deletion (range 3 to 84; *p* < 0.78). The mean age of patients who tested positive for a pathologic *PMP22* variant was 40 years (range 1 to 82 years) and 46 years for the ones tested negative (range 2 to 86 years; *p* < 0.04 for difference). Diagnosis of diabetes was reported in three patients with a pathologic variant of *PMP22* and in 20 without a variant (*p* < 0.14 for difference).

### Diagnostic yield in relation to testing indication

The highest diagnostic yield was observed when the testing was indicated by both the clinical presentation and suggestive family history with 38 (59%) of the total 64 positive findings (Table 2). Roughly half (55%) of the tests were performed because of the clinical presentation only. Among these, only 18% provided a diagnostic finding, 17% when testing for *PMP22* duplication and 20% when testing for the *PMP22* deletion. In the group 2 (positive family history) 15% of the performed tests had a diagnostic finding. In the group 3 (test based on both clinical features and positive family history), the overall diagnostic yield was highest (45%); 36% when testing for *PMP22* duplication, and 58% when testing for the *PMP22* deletion (Table 3). Diagnostic findings included 27 *PMP22* duplications (CMT1), 40 *PMP22* deletions (HNPP). In two individuals, a *PMP22* variant of unknown significance (c. 353 C > T, p.Thr118Met) was detected. Three *PMP22* deletions were identified when testing for *PMP22* duplication and were not considered diagnostic findings in testing for *PMP22* duplication.

**Table 2:** *PMP22* tests by indication and the distribution of diagnostic findings

	Group 1 (clinical) *N* (%)	Group 2 (family) *N* (%)	Group 3 (clin + fam) *N* (%)	Diagnostic findings in groups 1/2/3 (%)
PMP22 duplication	54 (47)	20 (17)	42 (36)	33 / 11 / 56
*PMP22* deletion	65 (59)	6 (5.4)	40 (36)	35 / 2.7 / 62
*PMP22* point mutation	15 (88)	0	2 (12)	0 / 0 / 0
Combined	134 (56)	26 (11)	84 (34)	34 / 6.3 / 59

Group 1: clinical presentation; Group 2: positive family history referring to confirmed diagnosis of CMT or HNPP in the family within two generations; Group 3: clinical presentation and suggestive family history

**Table 3:** Variants identified based on the testing indication

Variant	Group 1 (clin)	Group 2 (fam)	Group 3 (clin + fam)
*PMP22* duplication	9	3	15
*PMP22* deletion	13	1	23
*PMP22* point mutation	2	0	0
Combined	24	4	38

Groups by testing indication. Group 1: clinical presentation (clin); Group 2: positive family history (fam) referring to confirmed diagnosis of CMT or HNPP in the family within two generations; Group 3: clinical presentation and suggestive family history (clin + fam)

### Overview of identified *PMP22* variants

One patient diagnosed with clinical features suggesting CMT1A with an overlapping HNPP was identified in this study. Here, the genetic findings were a *PMP22* deletion together with a hemizygous (c. 353 C > T, p.Thr118Met) *PMP22* variant. Another patient was identified as harbouring the same p.Thr118Met variant in a heterozygous state, but here the clinical features were considered unconclusive and insufficient for a definite diagnosis of either CMT1 or HNPP. The patient presented with peroneus paresis after short-term impingement with normal ENMG findings with the symptoms mostly resolving over time. 

Recommendations for further genetic counselling were given, which the patient declined. The testing indication for both was clinical condition.

### Annual incidence and minimum prevalence estimates

The yearly incidence of the genetically confirmed *PMP22* neuropathies in Southwest Finland during the study period was 0.89/100 000, (95 % confidence interval 0.025–3.7/100 000). The yearly incidence for CMT1A was estimated at 0.4/100 000 (95 % confidence interval 0.025–3.7:100 000) and for HNPP at 0.6/100 000 (95 % confidence interval 0.025–3.7:100 000). The minimum prevalence estimates for CMT1A and HNPP in Southwest Finland based only on the diagnoses reached during the study period were 5.8/100 000 and 8.5/100 000, respectively.

## Discussion

We investigated the use of *PMP22* gene testing in diagnostics of inherited neuropathy at TUH during a period of 16 years and scrutinised the features of *PMP22* associated neuropathies in this population. Though more than 130 genes have been associated with CMT (particularly CMT2), the genetic aetiology of CMT1A is more straightforward [4, 11]. The 1.5 Mb duplication on chromosome 17p11.2 including the *PMP22* gene is responsible for the majority of CMT1A cases, representing the most common subtype of the most common inherited peripheral neuropathy. Deletion of the same locus is found in 85–90% of patients with clinical evidence of HNPP [4].

In this study, the diagnostic yield was 33% for *PMP22* deletions and 23% for *PMP22* duplications. Similarly, the *PMP22* duplication was found in 22% of patients suspected of CMT in a previous study [14]. The highest diagnostic yield (45%) for *PMP22* testing was reached when the testing was based on both the clinical presentation as well as positive family history. Notably, the diagnostic yield for testing for *PMP22* deletion (58%) was higher than that of *PMP22* duplication (36%) despite similar numbers of performed tests (40 and 42, respectively). Patients who tested negative for *PMP22* duplication may have other genetic subtypes of CMT, which can be diagnosed with further genetic testing utilizing e.g. NGS based targeted gene panels and whole exome sequencing. However, the eventual further genetic investigations beyond *PMP22* were not scrutinised in this study.

When the *PMP22* genetic testing was based on clinical presentation only (i.e. with no positive family history), the yield was 17% for *PMP22* duplication and 21% for *PMP22* deletion. *De novo* duplication and deletion variants of *PMP22* are reported with rates of 10% and 20%, respectively [4]. The findings of our study align with the current suggestion to conduct genetic testing of the *PMP22* gene even without a positive family history [2, 4]. In several previous studies, only patients already clinically diagnosed with CMT or HNPP were assessed for the presence of *PMP22* gene variants [10, 15]. This difference in approach creates some challenges when comparing our findings to previously reported data.

When the *PMP22* testing was based on confirmed diagnosis of a *PMP22* associated disorder in a family member, but there were no clinical symptoms clearly suggestive of CMT1 or HNPP, 15% of the tests for *PMP22* duplication and 17% for *PMP22* deletion resulted in a diagnostic finding. Given the dominant inheritance pattern of both disorders and near 100% penetrance of CMT1A, our findings lend support for genetic testing even in asymptomatic patients with a positive family history [2, 4]. The asymptomatic patients in our study with the *PMP22* duplication may well develop clinical symptoms later in life.

In our data, three diagnostic findings of *PMP22* deletion were made in patients initially suspected of having CMT. HNPP patients with a mistaken initial diagnosis of CMT have been reported [6]. This is probably explained by less typical phenotypes with features such as distal lower limb weakness, *pes cavus*, and diminished tendon reflexes that are shared by both disorders [4, 6].

Patients with a pathogenic variant in *PMP22* underwent genetic testing at mean six years younger than patients without a pathologic variant. This may reflect the earlier clinical onset of *PMP22*-associated neuropathies, with symptoms typically developing during the first two decades of life in CMT1A, and during the second or third decade in HNPP [4, 8]. Mean age at testing is higher than the average age at symptom onset, a common observation in slowly progressive genetic conditions with insidious symptom onset.

In two patients, a p.Thr118Met variant in *PMP22* was identified in gene sequencing. This variant has an ACMG classification of uncertain significance (3) [13]. The variant was interpreted as linked to a typical CMT1A phenotype in one patient. For the other patient, the finding was not considered diagnostic or interpreted as linked as the clinical phenotype was not considered typical for a *PMP22* associated neuropathy. The role of the p.Thr118Met variant in connection to CMT1 and HNPP is still unclear, and it is not considered definitively pathogenic [16–18].

### Comparison to national and global prevalence estimates

In our study, 27 *PMP22* duplications were identified, and the yearly incidence of CMT1A was 0.4:100 000. The minimum prevalence estimate for CMT1A, based on cases identified between 2005 and 2020, was 5.8:100 000. The global prevalence estimates of *PMP22* associated CMT1A range widely from 8.0:100 000 to 26:100 000 [4]. In the Finnish province of Northern Ostrobothnia, the estimated prevalence of all CMT has been reported at 35:100 000, while the prevalence of CMT1 was 6.6:100 000, with CMT1 accounting for 19% of all CMT cases [19]. In northern Sweden, the prevalence of CMT1 has been reported at 16:100 000 [20, 21]. Our minimum prevalence estimate is thus lower than what is commonly reported worldwide, but more comparable to the prevalence previously reported in the other Finnish region of Northern Ostrobothnia. For HNPP, the incidence of *PMP22* deletion was 0.6:100 000, and the minimum prevalence estimate was 8.5:100 000 based on diagnosed cases in this study. In comparison, a higher prevalence (16:100 000) for HNPP in Southwest Finland was reported in another study 20 years ago [6]. Both figures are, however, in line with the previously reported global prevalence estimate of 7.3:100 000 to 16:100 000 [4].

### Strengths and limitations

The strengths of the present study include access to individual EMR data and thus the details of clinical features, reported family history, and the final diagnoses of the patients. For the study period of 16 years, we can provide a good evaluation on the clinical use of targeted *PMP22* gene testing and the incidence of *PMP22* associated neuropathy. As a weakness, we acknowledge that as we could not include data on CMT1A or HNPP diagnoses from before 2005, our prevalence figures are likely quite conservative minimum estimates.

Since the testing was carried out over a long period, and by several clinicians, we were unable to reconstruct the precise reasoning behind the testing strategy chosen for individual patients based on patient records. In this study, the total number of NGS studies was low (17), and these were mostly performed by various commercial providers of diagnostic genetic panel services in Finland and elsewhere in the EU. In more recent years (2020s), most NGS panels at TUH are provided by an in-house genetic diagnostic service.

In our study on *PMP22* variants associated with both CMT1 and HNPP phenotypes, a significant proportion of diagnostic findings (37 out of 64, 58%) were *PMP22* deletions. Majority of the identified *PMP22* connected genetically confirmed diagnoses were of HNPP (40 out of 67, 60%). There are relatively few studies that have focused on HNPP compared to the extensive literature on CMT. The population prevalence of HNPP is not well known and has been probably underestimated because of the large clinical variability associated with HNPP. Moreover, oligosymptomatic patients might not seek treatment and thus remain undiagnosed [4, 6, 22].

### Conclusions

We find that in addition to clinical features suggestive of a *PMP22* associated neuropathy, a positive family history considerably increases the diagnostic yield of genetic testing. The diagnostic yield of testing for *PMP22* deletion and the overall proportion of HNPP diagnoses suggest that the prevalence, diagnostics, and clinical spectrum associated with HNPP merits more research attention. 

## Data Availability

The data that support the findings of this study are not openly available due to reasons of sensitivity and are available from the corresponding author upon reasonable request. Data are located in controlled access data storage at Turku University Hospital.
